# Myricetin exposure reduces PC differentiation in vitro in primary human B cells

**DOI:** 10.1186/s10020-025-01068-x

**Published:** 2025-01-29

**Authors:** Shabirul Haque, Betty Diamond

**Affiliations:** Center for Autoimmune Musculoskeletal and Hematopoietic Diseases, Institute of Molecular Medicine, The Feinstein Institutes for Medical Research, Northwell Health, 350 Community Drive, Manhasset, New York 11030 USA

**Keywords:** Myricetin, Flavonoids, B cell differentiation, Plasma cells, SLE

## Abstract

**Background:**

The process of B cell activation and plasma cell (PC) formation involves morphological, transcriptional, and metabolic changes in the B cell. Blocking or reducing PC differentiation is one approach to treat autoimmune diseases that are characterized by the presence of pathogenic autoantibodies. Recent studies have suggested the potential of myricetin, a natural flavonoid with anti-inflammatory and antioxidant properties, to block or reduce PC differentiation.

**Methods:**

Primary human B cells were purified by using a human B cell isolation kit. B cell subsets such as IgG memory B cells, marginal zone B cells (MZ B cells), and naive B cells were isolated by flow cytometry and activated to induce PC differentiation. Quantification of PCs (CD27 + + , CD38 +) was obtained by flow cytometry. The expression of mRNA was measured by qPCR. Ig secretion in culture supernatant was measured by ELISA.

**Results:**

Myricetin treatment significantly reduced PC differentiation in primary human B cells and all B cell subsets. Myricetin exposure reduced Ig production both IgM and IgG, in culture supernatants at day 5. Myricetin treatment led to augmented BACH2 expression and reduced IRF4, BLIMP1, and XBP1 expression compared to control cultures.

**Conclusion:**

Myricetin treatment reduced PC differentiation and Ig secretion by primary human B cells. Targeting B cells in this way may be a therapeutic approach for some autoimmune diseases.

**Graphical Abstract:**

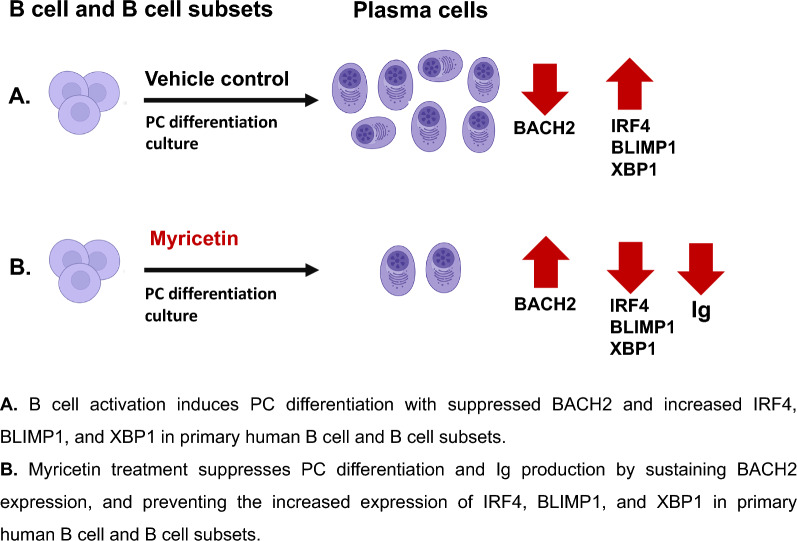

**Supplementary Information:**

The online version contains supplementary material available at 10.1186/s10020-025-01068-x.

## Introduction

Several autoimmune diseases such as systemic lupus erythematosus (SLE) are characterized by the presence of pathogenic autoantibodies produced by plasma cells (PCs). PCs generate protective antibodies during infection, but they also can produce pathogenic autoantibodies (Malkiel et al. 2018). Therapeutic approaches to reduce or block PC differentiation for the treatment of autoimmune diseases such as co-stimulatory blockade, cytokine blockade and proteasome blockade with limited success due to insufficient efficacy or too much toxicity have been explored (Atisha-Fregoso et al. 2021). There are several transcription factors which are involved in PC differentiation, such as IRF4, BLIMP1, and XBP1. The expression of these genes requires downregulation of PAX5, BACH2, and BCL6. BACH2 is a pivotal player in PC differentiation, as a high level of BACH2 represses transcription of BLIMP1 and IRF4 (Huang et al. 2014; Itoh-Nakadai et al. 2017; Hu et al. 2022; Ochiai et al. 2006; Ochiai et al. 2008; Ochiai et al. 2024) and BACH2 overexpression suppresses PC differentiation in mouse B cells in vivo (Finney and Kelsoe 2021). BACH2 deficient mice develop a lupus like-autoimmune disease.

Myricetin, a natural flavonoid, has been studied in various diseases and cell types. Myricetin treatment upregulates BACH2 expression. Elevated BACH2 expression in cardiomyocytes leads to downregulation of Akaf6, Bnp, and Myh7 all involved in cardiac hypertrophy in vivo (Jiang et al. 2022) and administration of myricetin improves autoimmune myocarditis in mice. Natural flavonoids particularly quercetin, rutin, myricetin, luteolin, enhance the efficacy of a therapeutic regimen in human lupus patients (Rh 2004). The study of myricetin in human PC differentiation has not been investigated.

We show here that myricetin reduces B cell proliferation PC differentiation and Ig production in primary human B cells and all B cell subsets.

## Materials and methods

### Reagents

Cell culture plates (96 well U-bottom, REF: 351177, polystyrene non pyrogenic), Iscove’s Modified Dulbecco’s Medium (cat# 12440-053, Invitrogen), fetal calf serum (cat# SH30070.03 Hyclone, Thermo Scientific), penicillin/streptomycin (cat# 15070063, Thermo Fisher Scientific), Myricetin (cat# CAS 529-44-2 Calbiochem, Millipore Sigma) dissolved in DMSO (cat# 276855, Sigma-Aldrich). The vehicle control (VC) (VC) for myricetin was DMSO. R848/Resiquimod (cat# tlrl-r848, InvivoGen) working concentration 2.5 µg/mL, CpG/ODN2006 (cat# tlrl-2006, InvivoGen) working concentration 1.0 µM, anti-human IgM antibody (cat# 2020-01, southern biotech) working concentration 1.0 µg/mL, IL-2 (Peprotech 200-02) working concentration 50.0 ng/mL, IL-10 (cat# 217-IL-010, R&D systems) working concentration 250.0 ng/mL, IL-21 (Peprotech 200-21) working concentration 25.0 ng/mL. MEGACD40L protein soluble (cat# ALX-522-110-C010, Enzo) working concentration 200.0 ng/mL.

### Antibodies

B cells were stained with following antibodies. PE/Cyanine 7 anti-human CD19 (cat# 302216, clone-HIB19, Biolegend), Anti-hu CD27-PE, eBioscience (cat# 12-0279-42, clone-0323, Invitrogen), Anti-hu CD38-PE Texas Red eF610, eBioscience (cat# 61-0389-42, clone-HIT1, Invitrogen), IgA antibody anti-human, APC (cat# 130-113-472, clone-IS 11-8E10, Miltenyi Biotec), Brilliant violet 421/pacific blue, anti-human CD1c (cat# 331526, clone-L161, Biolegend), FITC mouse anti-human IgG (cat # 555786, BD Pharmingen), FVD-eF506 eBioscience (cat# 65-0866-14, Thermo Fisher Scientific), Brilliant violet 421/pacific blue, anti-human IgM (cat # 314516, clone-MHM-88, Biolegend), PE/Cyanine7 anti-human CD138 (Syndecan-1) antibody (cat # 352318, Biolegend).

### Isolation of PBMCs and B cell purification

Leukopaks were obtained from healthy adult donors (New York Blood Center, NY). Peripheral blood mononuclear cells (PBMCs) were isolated by density gradient centrifugation using Cytiva Ficoll-Paque PLUS (cat# 45-001-749, Fisher Scientific). B cells were purified utilizing EasySep^™^ Human B Cell Isolation Kit (cat# 17954, Stem Cell).

### Isolation of naïve B cells, marginal zone (MZ) B cells and IgG memory B cells

Purified B cells (10–20 × 10^6^ B cells) were stained for isolation of B cell subsets. B cells were washed (300 g for 5 min at 4 °C) with washing buffer (5% FBS in HBSS). A cocktail of conjugated antibodies was prepared. For naïve and marginal zone B cell isolation, antibody cocktails were the following (CD19-PECy7, 1:50; CD27-PE, 1:50; CD38-PE Texas Red eF610, 1:50; IgA-APC, 1:100; CD1c-BV421/pacific blue,1:20; IgG-FITC, 1:25; FVD-eF506, 1:500). For IgG memory B cell isolation, antibody cocktails were the following (CD19-PECy7, 1:50; CD27-PE, 1:50; CD38-PE Texas Red eF610, 1:50; IgA-APC, 1:100; IgM-BV421/pacific blue, 1:100; FVD-eF506, 1:500). Cells and antibodies cocktail were mixed and incubated on ice for 30 min in dark. Cells were washed twice with 5% FBS in HBSS and resuspend cells in 5% FBS HBSS for B cell subsets sorting. Cell sorting was carried out at high pressure by BD FACSAria^™^ Cell Sorter machine.

### Naïve B cell activation for PC differentiation

Naïve B cells were stimulated for PC differentiation following Marsman C et al. (Marsman et al. 2022) method with minor adjustment. Briefly, 10,000 cells/well CD40L low cells (Luo et al. 2009) were plated in 96-well culture plate 24 h prior to naïve B cell addition in 10% FBS in IMDM medium. The next day, plates were washed with culture medium and sorted naïve B cells were added to the plate containing IL-21 (50.0 ng/mL, Peprotech). Naïve B cells were treated with VC or myricetin (200 µM) and cultured for PC differentiation for the period of 6 days. On day 2, anti-CD40L antibody (13.0 ug/mL) (cat # 100-1352, clone 5C8, stem cell) was added to the culture for termination of CD40L signalling.

### Staining for PC detection

B cells were harvested from culture, washed with washing buffer (5% FBS in HBSS) and centrifuged at 300 *g* for 5 min at 4 °C. Cells were stained with conjugated antibodies in washing buffer (CD19-PECy7, 1:50; CD27-PE, 1:50; CD38-PE/eF610, 1:50; FVD-eF506, 1:500) on ice for 30 min in the dark. Cells were washed twice, and cell pellets were re-suspended in 1% paraformaldehyde in HBSS. Re-suspended cells were analyzed by flow cytometry.

### Gene expression by qPCR

Total RNA was extracted from human B cells and B cell subsets using RNA extraction kit (cat# R2062, Direct-zol, RNA MicroPrep, ZymoResearch). Total RNA was converted into cDNA using iScript cDNA synthesis kit (cat# 1708890, Bio-Rad). Total cDNA was subjected to PreAmp using TaqMan PreAmp master mix (cat# 4391128, Applied Biosystems) before q-PCR. Primers were obtained from Thermo Fisher Scientific, human BACH2 (hs00935338_m1), human IRF4 (hs00180031_m1), human BLIMP1 (hs001533_m1), human XBP1 (hs02856596_m1), and human IRE1a (hs00980095_m1), human PAX5 (Hs00277134_m1), human BCL6 (Hs00153368_m1). Human UBC (hs05002522_g1) was used as a housekeeping gene for qPCR.

### Quantification of IgG and IgM in culture supernatants by ELISA

ELISA plates (costar assay plate, 96 well clear, flat bottom, half area high binding, polystyrene, cat# 3690, Corning) were coated with goat anti-human IgG-UNLB (1:100 diluted in 1 × PBS, cat # 2040-01, Southern Biotech) or IgM-UNLB (1:500 diluted in 1 × PBS, cat # 2020-01, Southern Biotech), 25 µl/well and the plate was incubated at 4 °C overnight. The next day, the plate was washed four times with washing buffer (PBS 0.05% Tween-20) and blocked with blocking buffer (1% BSA in PBS) for 1 h at room temperature (RT) and plates were washed. Culture supernatants (fivefold diluted) from the B cell cultures were added to the wells (25 µl/well). Standard human IgG-UNLB (cat# 0150-01, Southern Biotech) or standard human IgM lambda-UNLB (cat# 0158L-01, Southern Biotech) was added in serial twofold dilutions from 200 to 0.2 ng/mL and 0.0 ng/mL to the plate to generate a standard curve and incubated for 2.0 h at RT. The ELISA plate was washed four times. Goat anti-human IgG-AP conjugated (cat # 2040-04, Southern Biotech) at 1:1000 dilution or goat anti-human IgM-AP (cat# 2020-04, Southern Biotech) at 1:500 dilution in 0.2% BSA in PBS were added to the wells. The plate was incubated for 1.0 h at room temperature and washed four times. Phosphatase substrate buffer was added (50 µl/well) and the plate was incubated until color developed at 37 °C in the dark (around 25 min was the optimum time for color development). Absorbance of each well was measured at 405 nm (Victor^3^, 1420 multilabel counter plate reader, Perkin Elmer).

### B cell proliferation assay

Purified B cells were labeled with CellTrace Violet utilizing cell proliferation kit (cat# C34557, Invitrogen) by following the manufacturer’s protocol. After labelling, B cells were cultured in 10% FBS in IMDM containing stimulants R848, IL2, IL10, and IL21 and either VC or Myricetin (200 µM). On day 3, B cells were harvested and stained with FVD eF660 (cat # 65-0864-14, Invitrogen/eBioscience). Stained cells were analyzed for cell proliferation by flow cytometry.

### Statistical analysis

To compare the mean values between two groups, a student’s t-test was used. Statistical significance was defined as p < 0.05.

## Results

### Myricetin reduces PC differentiation in primary human B cells

**Fig. 1:**
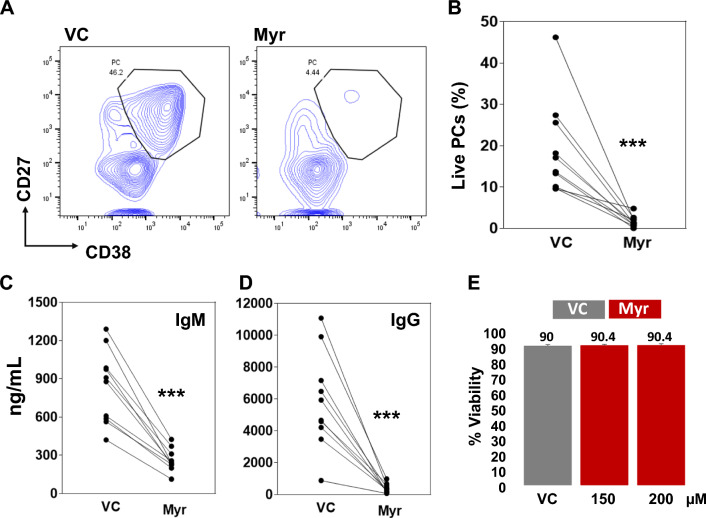
Myricetin reduces PC differentiation in primary human B cells. **A** Total B cells (n = 10 healthy donors) were activated with R848, IL-2, IL-10, IL-21 in the presence of myricetin (200 µM) or VC. On day 5, B cells were harvested and stained to detect PCs. **B** Cumulative data was plotted from B cells of all the donors. **C** and **D** IgM and IgG concentration was determined in day 5 culture supernatants by ELISA. **E** Effect of myricetin (150, and 200 µM) on cell viability was determined by cell counter in day 1 culture. P value was calculated between VC and Myr. ***p < 0.001

To test the effect of myricetin on PC differentiation, purified B cells were activated with R848, IL-2, IL-10, and IL-21 and cultured for 5 days. Myricetin (200 uM) or vehicle control (VC) was added to the culture at day 0. On day 5, cells were harvested and stained for PC markers (CD19 + , CD27 + + , CD38 + , live +). Myricetin exposure led to a significant reduction in the number of PCs compared to VC in seven different healthy donors (Fig. 1A, B). Culture supernatants contained less IgM and IgG when myricetin was present (Fig. 1C, D). Myricetin had no effect on cell viability (Fig. 1E).

**Fig. 2:**
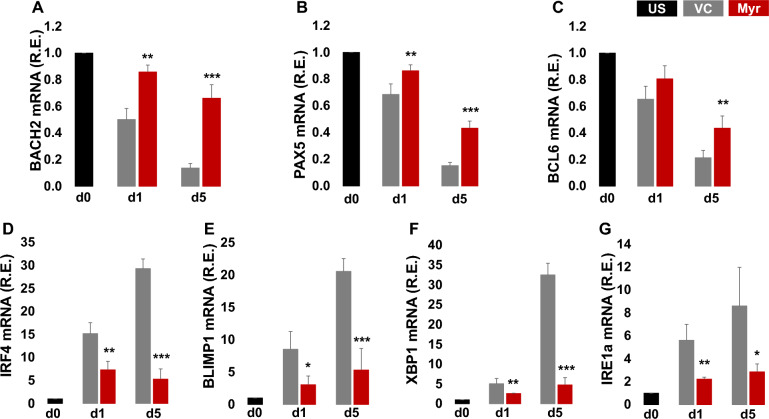
Myricetin led to sustained BACH2 expression, and diminished the increase in IRF4, BLIMP1, and XBP1 expression in primary human B cells. Total B cells (n = 3 healthy donors) were activated with R848, IL-2, IL-10, IL-21 in the presence of myricetin (200 µM) or VC. Total RNA was isolated from B cells at d0 (unstimulated t = 0), day 1 culture and day 5 culture as indicated. Relative gene expression of BACH2 (**A**), PAX5 (**B**), BCL6 (**C**), IRF4 (**D**), BLIMP1 (**E**), XBP1 (**F**), and IRE1α (**G**) was evaluated by qPCR. *p < 0.05, **p < 0.01, ***p < 0.001

To evaluate the response of myricetin on PC associated genes, BACH2, PAX5, BCL6, IRF4, BLIMP1, XBP1, and the ER stress gene IRE1a (Inositol-Requiring Enzyme 1a (IRE1a) which is responsible for XBP1 splicing. Purified B cells were activated with R848, IL-2, IL-10, and IL-21 and treated with myricetin (200 µM) or vehicle control (VC). B cells and culture supernatants were harvested on day 1 and day 5. Unstimulated B cells were harvested at time zero (d0). Activated B cells showed decrease in BACH2, PAX5, and BCL6 expression already on day 1 and day 5. Myricetin treated cells expressed more BACH2, PAX5, and BCL6 mRNA compared to VC treated cells on both day 1 and day 5 and sustained expression of BACH2, PAX5 and BCL6 comparable to unstimulated B cells as demonstrated BACH2 (Fig. 2A), PAX5 (Fig. 2B), BCL6 (Fig. 2C). B cell activation led to an increase in expression of IRF4 (Fig. 2D), BLIMP1 (Fig. 2E), XBP1 (Fig. 2F), and IRE1a (Fig. 2G), on day 1 and day 5 which did not occur to the same extent in myricetin treated cultures. Myricetin suppressed PC differentiation by maintaining BACH2 expression, thereby preventing IRF4, BLIMP1, XBP1, and IRE1a mRNA expression.

**Fig. 3:**
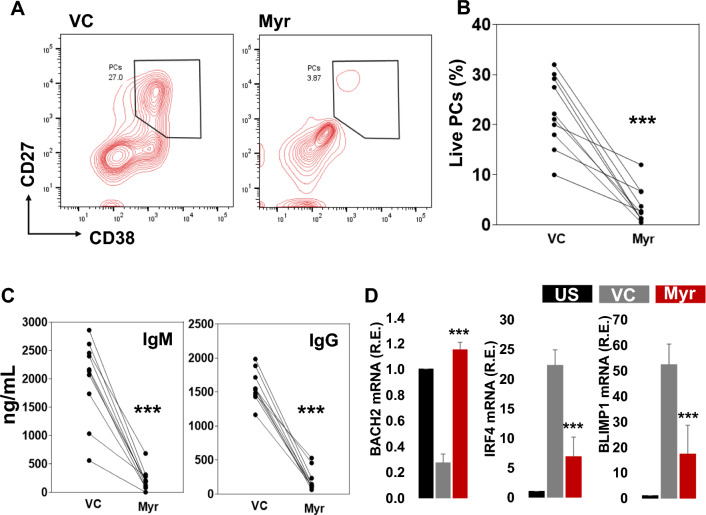
Myricetin reduces PCs in CD40L, CpG, IL10, IL21 activated B cells. **A** Total B cells (n = 10 healthy donors) were activated with CD40L, CpG, IL-10, and IL-21 in the presence of myricetin (200 µM) and vehicle control (VC) and cultured for PC differentiation. On day 5, cells were harvested and stained with CD19, CD27, and CD38 for live PC evaluation. **B** Overall, data was plotted from all the donors. **C** IgM and IgG concentration was estimated in day 5 culture supernatants by ELISA. **D** PC differentiation associated genes (BACH2, IRF4, and BLIMP1) expression was determined by qPCR. Relative expression was calculated using unstimulated (US) as control (black bar), VC (gray bar) and Myr (red bar). P value was calculated between VC and Myr. P value (*p < 0.05, **p < 0.01, ***p < 0.001)

We also activated B cells with CD40L, CpG, IL-10, and IL-21 and treated them with myricetin (200 µM) or VC. On day 5, cells were harvested and stained for PC detection. Myricetin exposure reduced PC differentiation in cultures of all donors (Fig. 3A, B). Myricetin reduced IgM and IgG production in culture supernatants (Fig. 3C). BACH2 expression was downregulated with activation compared to unstimulated B cells. Myricetin treatment led to an increase in BACH2 expression relative to VC treatment. IRF4 and BLIMP1 expression was augmented upon activation compared to unstimulated B cells, and significantly suppressed by myricetin exposure (Fig. 3D).

### Myricetin treatment reduced PC differentiation in all B cell subsets

To evaluate the response of individual B cell subsets to myricetin, naïve, marginal zone (MZ) and IgG memory B cells were isolated. Naïve B cells and MZ B cells were isolated by implementing the following gating strategy (Supplemental Fig. 1A). IgG memory B cells were isolated by implementing the following gating strategy (Supplemental Fig. 1B). We also checked the purity of each B cell subset after sorting. We found naïve B cells were 97.5% pure, MZB cells, 96.6%; and IgG memory B cells, 91.3% as shown in Supplemental Fig. 2.

**Fig. 4:**
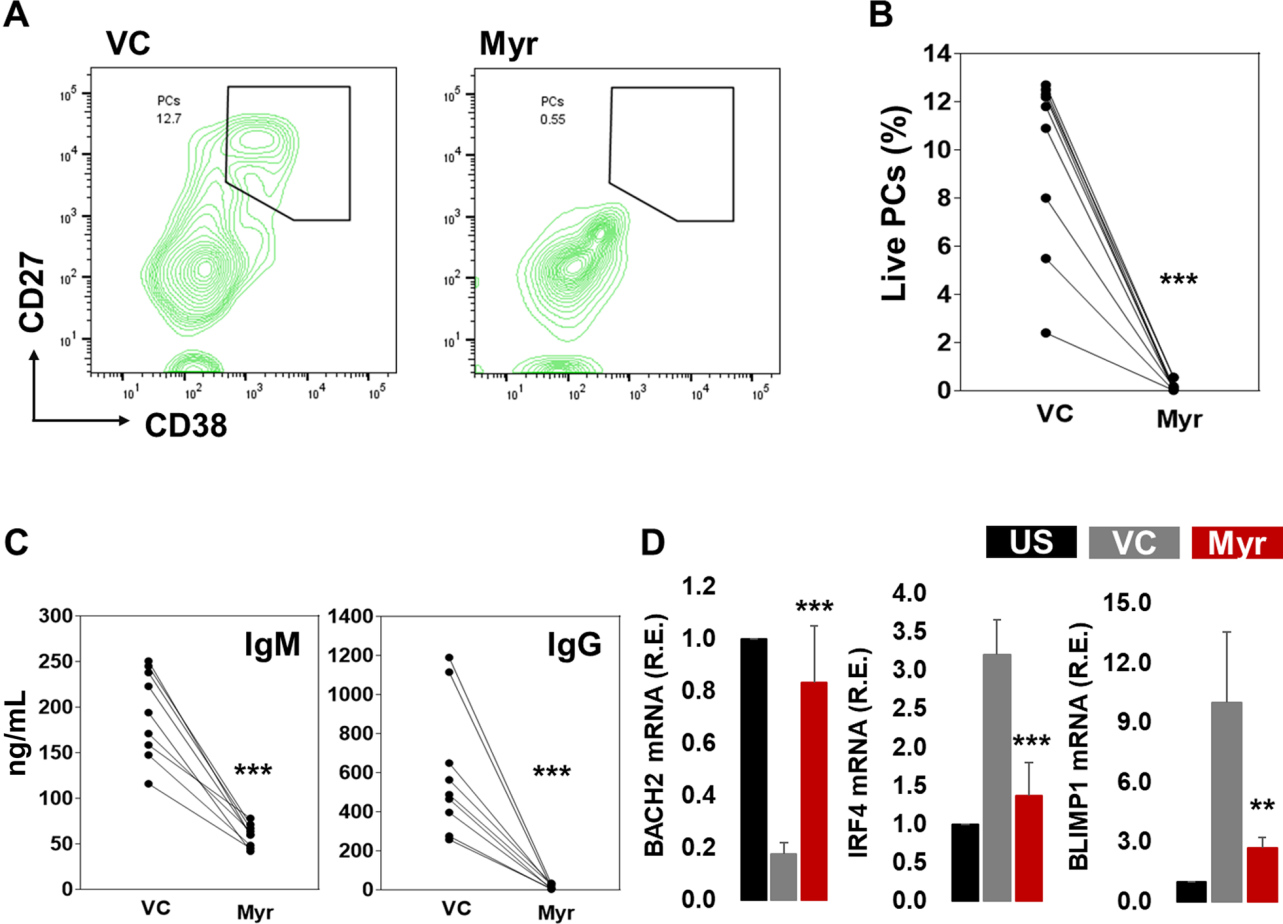
Myricetin exposure reduced PC differentiation in naive B cells. **A** Naive B cells (n = 9 healthy donors) were activated with IL-21, and CD40L expressing feeder cells and treated with myricetin (200 µM) or VC. On day 6, cells were harvested, and live PCs were evaluated by flow cytometry. **B** Cumulative data was plotted from all the donors. **C** IgM and IgG concentration was quantified in day 6 culture supernatants by ELISA. **D** PC related gene expression detection, naive B cells were activated with R848, anti-IgM, IL-2, IL-10, and IL-21 for 24 h and treated with Myr (200 µM) or VC, and gene (BACH2, IRF4, and BLIMP1) expression was quantified by qPCR. P value VC vs. Myr, **p < 0.01, ***p < 0.001

Naive B cells were activated for PC differentiation with IL-21, and anti-CD40L. On day 0, naïve B cells were treated with myricetin (200 uM) or VC. Myricetin treatment led to less PC differentiation on day 6 culture compared to VC (Fig. 4A, B). Myricetin also led to reduced IgM and IgG secretion in day 6 culture supernatants (Fig. 4C). Naive B cells were activated with R848, anti-IgM, IL-2, IL-10, and IL-21 for 24 h along with either myricetin or VC and gene expression was analyzed by qPCR. BACH2 expression was reduced from baseline in activated B cells, myricetin treatment maintained BACH2 expression. IRF4 and BLIMP1 expression was augmented with stimulation. Myricetin exposure prevented the increase on IRF4 and BLIMP1 expression (Fig. 4D).

**Fig. 5:**
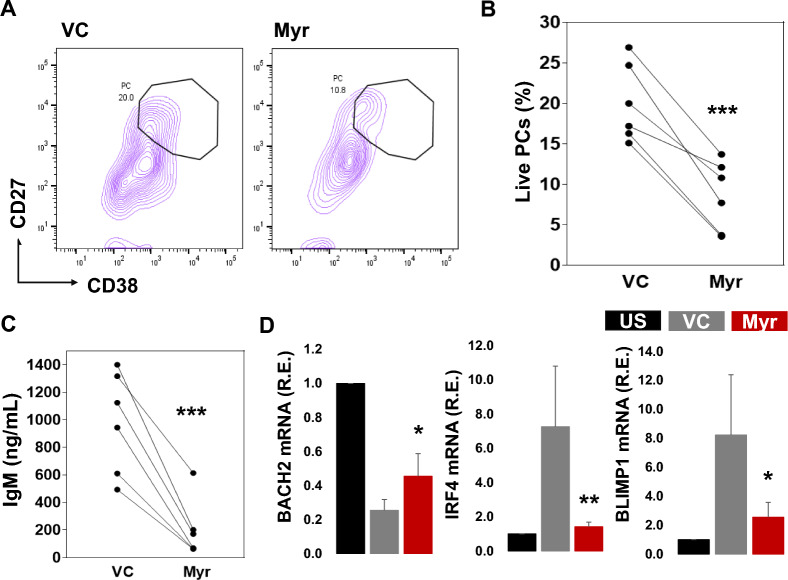
Myricetin exposure reduced PC differentiation in MZ B cells. **A** MZ B cells (n = 6 healthy donors) were activated with R848, IL-2, IL-10, IL-21 and treated with myricetin (200 µM) or VC and cultured. On day 5, cells were harvested, and live PCs were evaluated by flow cytometer. **B** Cumulative data was plotted from all the donors. **C** IgM concentration was quantified in day 5 culture supernatants by ELISA. **D** To study PC differentiation related gene expression, MZ B cells were activated with only R848 for 24 h then relative gene expression of BACH2, IRF4, and BLIMP1 was quantified by qPCR. Relative expression was calculated using unstimulated (US) as control sample (black bar) vs. VC (gray bar) and Myr (red bar). P value *p < 0.05, **p < 0.01, ***p < 0.001

MZ B cells were activated with R848, IL-2, IL-10, and IL-21 and treated with myricetin (200 µM) or VC. Myricetin treatment led to less PC differentiation on day 5 compared to VC treatment (Fig. 5A, B). Myricetin also led to reduced IgM secretion in day 5 culture supernatants (Fig. 5C). MZ B cells were activated for 24 h along with either myricetin or VC and gene expression was evaluated by qPCR. BACH2 expression was greater in myricetin treated cells than VC treated cells. IRF4 and BLIMP1 expression was augmented with stimulations, myricetin exposure prevented the increase in IRF4 and BLIMP1 expression (Fig. 5D).

**Fig. 6:**
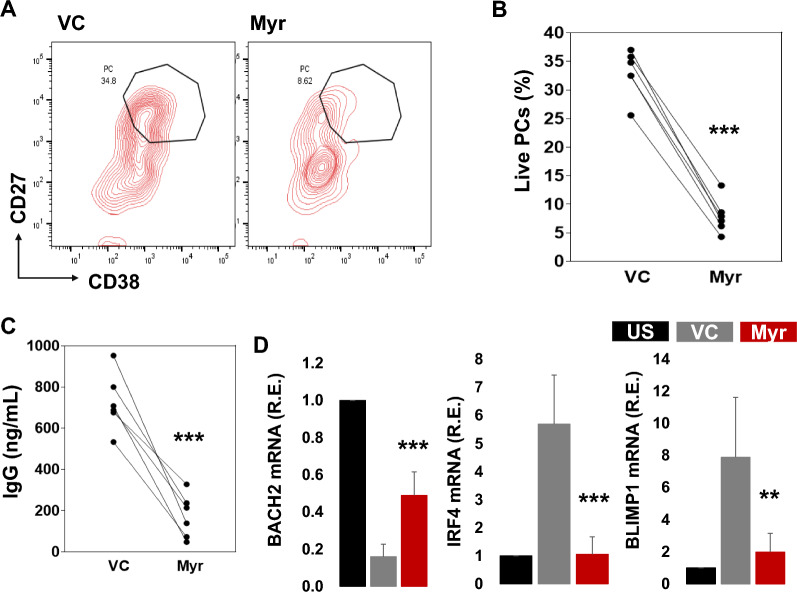
Myricetin treatment reduced PC differentiation in IgG memory B cells. **A** IgG memory B cells (n = 6 healthy donors) were activated with R848, IL2, IL-10, and IL-21 and treated with myricetin (200 µM) or VC. On day 5, cells were harvested and stained with CD19, CD27, CD38, and live dead dye, measured by flow cytometer. **B** Cumulative data was plotted. **C** IgG concentration was quantified within day 5 culture supernatants by ELISA. **D** IgG memory B cells were activated with R848, IL-10, and IL-21 for 24 h and gene (BACH2, IRF4, and BLIMP1) expression was evaluated by qPCR. Relative expression was calculated using unstimulated (US) as control sample (black bar) vs. VC (gray bar) and Myr (red bar). **p < 0.01, ***p < 0.001

IgG memory B cells were activated with R848, IL-2, IL-10, and IL-21 for PC differentiation and treated with myricetin (200 µM) or VC. Myricetin treatment reduced PC differentiation (Fig. 6A, B) on day 5 and led to reduced IgG secretion in day 5 culture supernatants compared to VC treated samples (Fig. 6C). To study PC differentiation associated genes, IgG memory B cells were activated for 24 h in the presence of either myricetin or VC and gene expression was evaluated by qPCR. There was a reduction in BACH2 expression at 24 h compared to unstimulated B cells, but myricetin led to a less pronounced reduction. Myricetin exposure suppressed the increase in IRF4 and BLIMP1 expression (Fig. 6D).

### Myricetin treatment reduced B cell proliferation

**Fig. 7:**
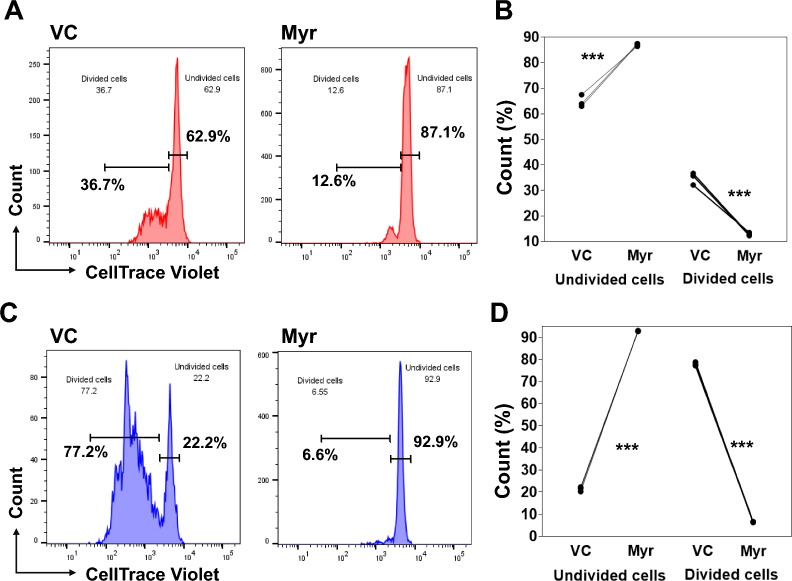
Myricetin treatment reduced B cell proliferation. B cells (n = 3 healthy donors) were labelled with CellTrace Violet. B cells were activated with R848, IL2, IL-10, and IL-21 and treated with myricetin (200 µM) or VC. B cells were harvested on day 3 & 5 and stained with FVD eF660. **A** and **B** shows cell proliferation status of day 3 cultured B cells. **C** and **D** shows cell proliferation status of day 5 cultured B cells. **B** and D shows cumulative data plotted from undivided and divided B cells from day 3 & 5 culture respectively. P value ***p < 0.001

To assess cell proliferation, B cells were labelled with CellTrace Violet. After labelling, B cells were activated with R848, IL-2, IL-10, and IL-21 and treated with myricetin (200 µM) or VC. Cell proliferation was assessed on day 3 and day 5. We found cell proliferation was reduced by myricetin compared to VC in day 3 cultured B cells (Fig. 7A, B) and day 5 cultured B cells (Fig. 7C, D). In both day 3 and day 5 cultured B cells, myricetin treatment increased the percentage of undivided cells and reduced the percentage of divided cells compared to VC treatment. Overall, myricetin functions as an antagonist to B cell proliferation.

### Myricetin treatment reduced IgM and IgG secretion in PCs

**Fig. 8:**
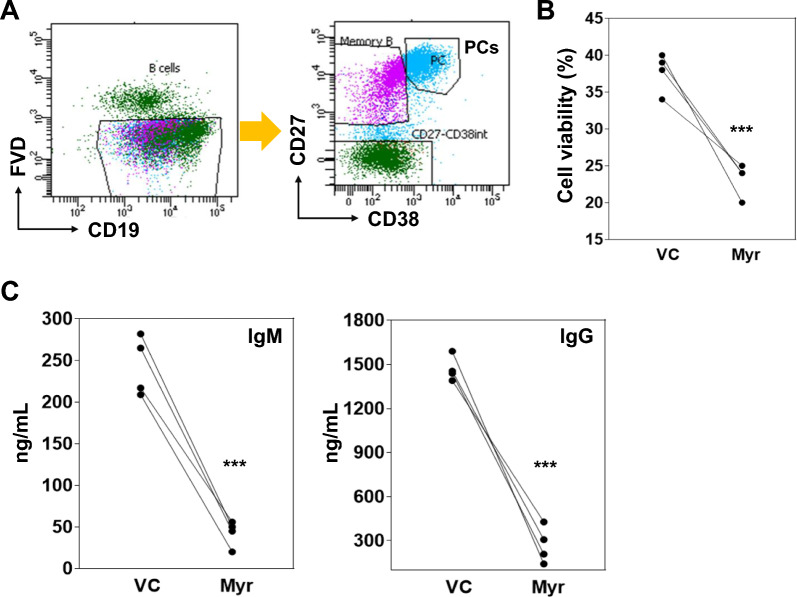
Myricetin treatment reduced Ig secretion by PCs. B cells (n = 4 healthy donors) were activated with R848, IL2, IL-10, and IL-21 for PC differentiation. **A** On day 5, PCs were sorted per gating strategy after staining with CD19, CD27, CD38, and live dead dye. **B** Sorted PCs were cultured in fresh culture medium containing stimulants and treated with either VC or myricetin (200 µM). Cell viability was measured after 24 h of treatment. **C** IgM and IgG concentration was quantified in culture supernatants of 24 h of treated conditions. P value was calculated between VC and myricetin group. **p < 0.01, ***p < 0.001

Total B cells (n = 4 healthy donors) were activated and differentiated with R848, IL-2, IL-10, and IL-21 and for 5 days. On day 5, the culture was harvested, and PCs were sorted per the gating strategy (Fig. 8A). Sorted PCs were treated with myricetin (200 µM) or VC and cultured in fresh cultured medium containing same stimulants. After 24 h, cell viability was quantified. Considering VC as 100% cell viability, in myricetin treated culture cell viability was ~ 60% (Fig. 8B). These data suggest PCs are susceptible to death during myricetin exposure. IgM and IgG was quantified in culture supernatants at 24 h. Considering the Ig concentration in the culture VC as 100% IgM and IgG production, myricetin treatment produced only 35% IgM and 37% IgG per cell (Fig. 8C). These data show myricetin exposure reduces Ig secretion.

## Discussion 

Myricetin treatment significantly reduced PC differentiation in all human B cell subsets and reduced Ig production (IgM and IgG) in culture supernatants. Myricetin treatment led to high BACH2 expression preventing PC differentiation, the upregulation of IRF4, BLIMP1, and XBP1 expression needed for PC differentiation.

Myricetin (3,3′,4′,5,5′,7‐hexahydroxyflavone) is a natural flavonoid, first extracted as yellow color crystals from the bark of Myrica nagi and was used as dye. In 1924, Jan Kalff and Robert Robinson (Kalff and Robinson 1925; Perkin and Hummel 1896) synthesized it. In last two decades, myricetin has been widely researched for its therapeutic potential in cancer, diabetes, and neurodegeneration (Büchter et al. 2013; Maggiolini et al. 2005; Ong and Khoo 2000). Myricetin targets multiple biological pathways. It inhibits activation of NF-kB, reduces ER stress, and induces NRF2/HO-1, an oxidative stress reducer.

In B cells, inhibition of NF-kB activation decreases PC differentiation (Guldenpfennig et al. 2023). Myricetin is a potent NF-kB inhibitor, in primary mouse macrophages and the RAW264.7 macrophage cell lines activated by LPS (Kang et al. 2005; Cho et al. 2016). PCs experience a high level of ER stress as immunoglobulin (heavy and light) chains are folded and assembled into biologically functional antibodies. Inositol-Requiring Enzyme 1a (IRE1a) and downstream X-Box Binding Protein 1 (XBP1) are needed for expansion of endoplasmic reticulum and secretion of antibodies (Shaffer et al. 2004). Ablation of XBP-1 expression significantly inhibits PC differentiation in mice and decreases antibody secretion (Tirosh et al. 2005; Todd et al. 2009). IRE1a KO, XBP1 KO, and IRE1a-XBP-1 double knockout mice all showed a reduction in PC differentiation and antibody secretion (Benhamron et al. 2014). BI09 an inhibitor of IRE1a which inhibits XBP1 splicing, prevents B cells from differentiating into PC, and decreases autoantibody production (Zhang et al. 2021). Myricetin treatment suppressed both XBP-1 and IRE1a mRNA expression in primary human B cells. Myricetin directly binds to human IRE1a leading to its degradation (Wiseman et al. 2010).

Nuclear factor erythroid 2-related factor 2 (NRF2), also known as NFE2L2, is a transcription factor that regulates the expression of genes that help to protect cells from damage from oxidative stress. NRF2-deficient mice develop antibodies against dsDNA and Sm, and exhibit deposition of IgG, IgM, C3, in kidney, heart and brain (Li et al. 2004). In MRL/lpr mice, expression of NRF2 is found to be low while expression of HMGB1, TLR, and NF-kB proteins are high. Adenovirus based NRF2 overexpression reduced HMGB1, TLR4, NF-kB and its downstream inflammatory cytokines (IL-1β and TNFα) expression (Li et al. 2023). The NRF2 inducer sulforaphane was demonstrated to have an anti-arthritis effect by inhibiting PC differentiation and production of inflammatory cytokines (IL-6, IL-17, TNFα etc.) (Moon et al. 2021). The NRF2 inducer 4-Octyl itaconate inhibited pro-inflammatory cytokines (IL-6, IL-1b, TNFα) in PBMC of SLE patients (Tang et al. 2018). In cardiomyocytes also, myricetin treatment augmented the activity and expression of NRF2 and HO-1 pathway and showed antioxidative properties by decreasing inflammatory cytokines (IL-6, IL-1β, TNFα) (Liao et al. 2017). Similarly, myricetin treatment helped to prevent the streptozotocin induced-diabetes mellitus through augmenting NRF2 (Yang et al. 2019).

## Conclusions

Myricetin exposure reduced PC differentiation and Ig production by inducing sustained expression of BACH2 in B cells. Thus, myricetin or its analogs might be a novel therapeutic for PC reduction in autoimmune diseases.

## Supplementary Information

Supplementary material 1: Figure 1 Gating strategy for IgG memory B cell, and Naïve B cell, and MZB cell sorting. Purified B cells were stained as mentioned in material and method section. A. Naïve B cells (CD19+, IgA-, IgG-, CD38 Int, CD27- B cell population) and MZ B cells (CD19+, CD27+, CD1c+, IgG-, IgA-, CD38Int B cell population) were sorted as per gating. B. IgG memory B cells (CD19+, CD27+, IgA-, IgM-, CD38 Int B cell population) were sorted as per gating. Figure 2 Purity of sorted B cell subsets. A. Naïve B cells were 97.5% pure. B. MZ B cells were 96.6% pure. C. IgG memory B cells were 91.3% pure.

## Data Availability

No datasets were generated or analysed during the current study.

## Abbreviations

BACH2: BTB domain and CNC homolog 2
IRF4: Interferon regulatory factor 4
BLIMP1: B lymphocyte-induced maturation protein-1
XBP1: X-box binding protein 1
SLE: Systemic lupus erythematosus
