# Supplementary material for: Myricetin exposure reduces PC differentiation in vitro in primary human B cells
Source: Mol Med. 2025 Jan 29;31:28. doi: 10.1186/s10020-025-01068-x (PMC11776280; doi:10.1186/s10020-025-01068-x)
Supplement: Supplementary file 1 — Supplementary material 1: Figure 1 Gating strategy for IgG memory B cell, and Naïve B cell, and MZB cell sorting. Purified B cells were stained as mentioned in material and method section. A. Naïve B cells (CD19+, IgA-, IgG-, CD38 Int, CD27- B cell population) and MZ B cells (CD19+, CD27+, CD1c+, IgG-, IgA-, CD38Int B cell population) were sorted as per gating. B. IgG memory B cells (CD19+, CD27+, IgA-, IgM-, CD38 Int B cell population) were sorted as per gating. Figure 2 Purity of sorted B cell subsets. A. Naïve B cells were 97.5% pure. B. MZ B cells were 96.6% pure. C. IgG memory B cells were 91.3% pure. [file 10020_2025_1068_MOESM1_ESM.pptx]

## Slide 1
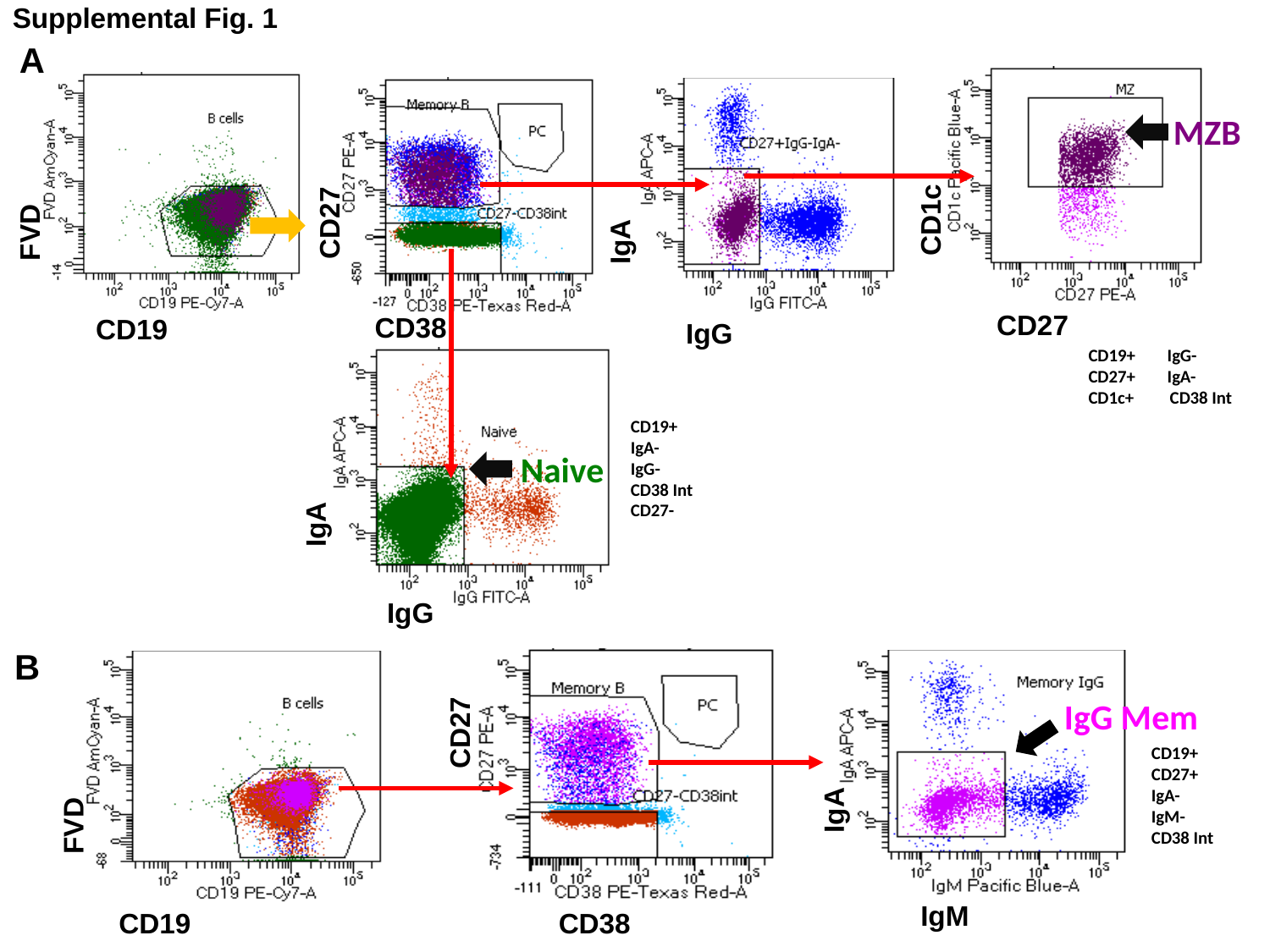

Supplemental Fig. 1
A
FVD
CD19
MZB
CD1c
CD27
IgA
CD27
CD38
IgG
CD19+ IgG-
CD27+ IgA-
CD1c+ CD38 Int
CD19+
IgA-
IgG-
CD38 Int
CD27-
Naive
IgA
IgG
B
IgG Mem
CD27
CD19+
CD27+
IgA-
IgM-
CD38 Int
IgA
FVD
IgM
CD19
CD38

## Slide 2
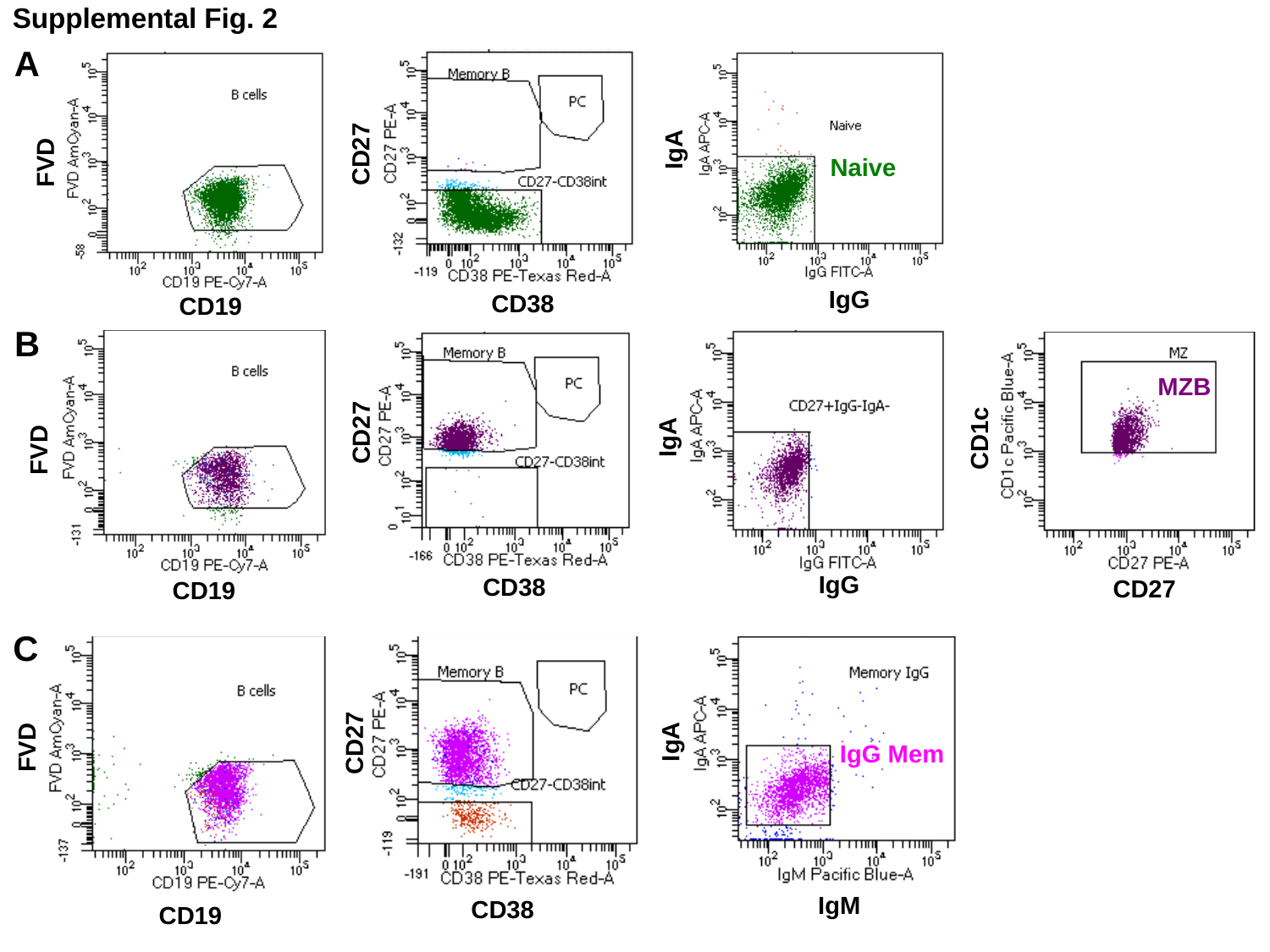

Supplemental Fig. 2
A
IgA
CD27
FVD
Naive
IgG
CD38
CD19
B
MZB
IgA
CD27
CD1c
FVD
IgG
CD38
CD27
CD19
C
IgA
FVD
CD27
IgG Mem
IgM
CD38
CD19
